# Supplementary figures and images for: Immunohistochemical analyses reveal FoxP3 expressions in spleen and colorectal cancer in mice treated with AOM/DSS, and their suppression by glycyrrhizin
Source: PLoS One. 2024 Aug 16;19(8):e0307038. doi: 10.1371/journal.pone.0307038 (PMC11329161; doi:10.1371/journal.pone.0307038)

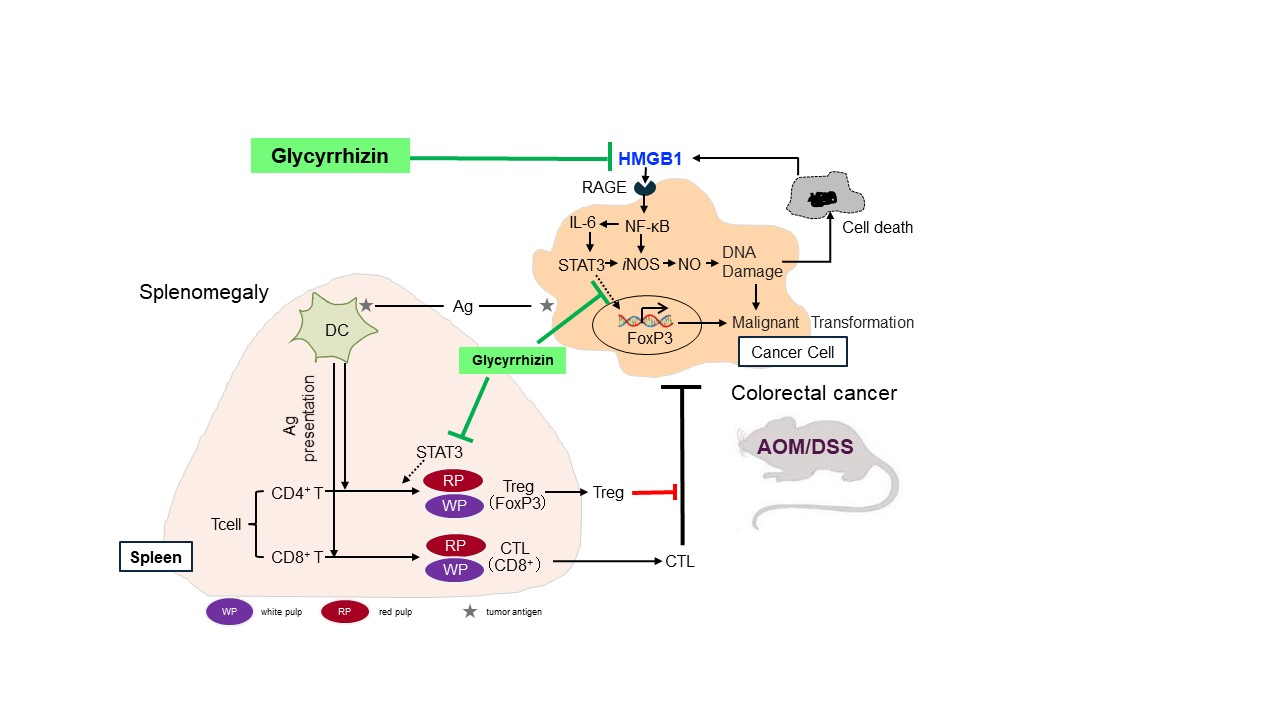

Supplement: S1 Graphical abstract — (TIF) [file pone.0307038.s007.tif]
