# Supplementary material for: Immunohistochemical analyses reveal FoxP3 expressions in spleen and colorectal cancer in mice treated with AOM/DSS, and their suppression by glycyrrhizin
Source: PLoS One. 2024 Aug 16;19(8):e0307038. doi: 10.1371/journal.pone.0307038 (PMC11329161; doi:10.1371/journal.pone.0307038)
Supplement: Attachment — Submitted filename: Response to Reviewers.docx [file pone.0307038.s008.docx]

June 12, 2024

Prof. Vinay Kumar,

Academic Editor

*PLOS ONE*

Dear Prof. Vinay Kumar,

We are pleased to resubmit the research article for consideration for publication in *PLOS ONE.* We deeply appreciate your and the Reviewers' critical reading of our manuscript PONE-D-24-15025 entitled “Immunohistochemical analyses reveal FoxP3 expressions in spleen and colorectal cancer in mice treated with AOM/DSS, and their suppression by glycyrrhizin” by Wang et al.

According to the Reviewer's comments, we extensively revised our manuscript and made much effort in improving the overall quality of our manuscript. We would like to answer the several comments proposed by the Reviewers, and explain the revised version in detail. Our replies are listed on a separate sheet.

Conflicts of Interest / COI statement

Author N.Y. was employed by a company Cokey Co., Ltd., operating licorice-related business. N.Y. 's position, etc. does not alter our adherence to PLOS ONE policies on sharing data and materials. Also, there are no restrictions from Cokey Co. Ltd, on all steps of this study. The remaining authors declare that the research was conducted in the absence of any commercial or financial relationships that could be construed as a potential conflict of interest.

We believe that the manuscript has been improved satisfactorily and hope that it will be accepted for publication in *PLOS ONE.*

Looking forward to hearing your decision at your earliest convenience.

Yours sincerely,

Mariko Murata M.D., Ph.D., Professor

Department of Environmental and Molecular Medicine,

Mie University Graduate School of Medicine,

2-174, Edobashi, Tsu, Mie, 514-8507, Japan.

Tel. /fax: +81-59-231-5011.

E-mail address: mmurata@med.mie-u.ac.jp

Journal Requirements:

When submitting your revision, we need you to address these additional requirements.

1. Please ensure that your manuscript meets PLOS ONE's style requirements, including those for file naming. The PLOS ONE style templates can be found at

<https://journals.plos.org/plosone/s/file?id=wjVg/PLOSOne_formatting_sample_main_body.pdf>

and

<https://journals.plos.org/plosone/s/file?id=ba62/PLOSOne_formatting_sample_title_authors_affiliations.pdf>

**Reply:** We have adapted our manuscript to meet PLOS ONE's style requirements, including those for file naming.

2. To comply with PLOS ONE submissions requirements, in your Methods section, please provide additional information regarding the experiments involving animals and ensure you have included details on (1) methods of sacrifice, (2) methods of anesthesia and/or analgesia, and (3) efforts to alleviate suffering.

**Reply:** We added the statements in the method section of our manuscript (Lines 95-97).

3. As part of your revision, please complete and submit a copy of the Full ARRIVE 2.0 Guidelines checklist, a document that aims to improve experimental reporting and reproducibility of animal studies for purposes of post-publication data analysis and reproducibility: https://arriveguidelines.org/sites/arrive/files/documents/Author%20Checklist%20-%20Full.pdf Please include your completed checklist as a Supporting Information file. Note that if your paper is accepted for publication, this checklist will be published as part of your article

**Reply:** We included the completed check list as a Supporting Information file.

4. We note that you have included the phrase “data not shown” in your manuscript. Unfortunately, this does not meet our data sharing requirements. PLOS does not permit references to inaccessible data. We require that authors provide all relevant data within the paper, Supporting Information files, or in an acceptable, public repository. Please add a citation to support this phrase or upload the data that corresponds with these findings to a stable repository (such as Figshare or Dryad) and provide and URLs, DOIs, or accession numbers that may be used to access these data. Or, if the data are not a core part of the research being presented in your study, we ask that you remove the phrase that refers to these data.

**Reply:** We changed the analytical method according to the reviewer’s comment, and we showed all data, and provided all relevant data within the paper, as Supporting Information files.

5. Thank you for stating the following financial disclosure:

"This study was partly supported by JSPS KAKENHI under Grant (numbers 19K10585 and 20K20320 to S.K. and 22H03331 to M.M.). This study was supported in partly by Cokey Co., Ltd. Under Grant (number 20190401 for N.Y.)."

Please state what role the funders took in the study. If the funders had no role, please state: "The funders had no role in study design, data collection and analysis, decision to publish, or preparation of the manuscript."

If this statement is not correct you must amend it as needed.

Please include this amended Role of Funder statement in your cover letter; we will change the online submission form on your behalf.

**Reply:** We added the statement "The funders had no role in study design, data collection and analysis, decision to publish, or preparation of the manuscript.".

6. Thank you for stating the following in the Competing Interests/Financial Disclosure * (delete as necessary) section:

"Conflicts of Interest / COI statement

Author N.Y. was employed by the company Cokey Co., Ltd. The remaining authors declare that the research was conducted in the absence of any commercial or financial relationships that could be construed as a potential conflict of interest."

We note that you received funding from a commercial source: "Cokey Co., Ltd"

Please provide an amended Competing Interests Statement that explicitly states this commercial funder, along with any other relevant declarations relating to employment, consultancy, patents, products in development, marketed products, etc.

Within this Competing Interests Statement, please confirm that this does not alter your adherence to all PLOS ONE policies on sharing data and materials by including the following statement: "This does not alter our adherence to PLOS ONE policies on sharing data and materials.” (as detailed online in our guide for authors http://journals.plos.org/plosone/s/competing-interests). If there are restrictions on sharing of data and/or materials, please state these. Please note that we cannot proceed with consideration of your article until this information has been declared.

Please include your amended Competing Interests Statement within your cover letter. We will change the online submission form on your behalf.

**Reply:** We added the statement about COI of the author N.Y. with Cokey Co. Ltd. in both COI statement in the manuscript and cover letter.

7. Your ethics statement should only appear in the Methods section of your manuscript. If your ethics statement is written in any section besides the Methods, please move it to the Methods section and delete it from any other section. Please ensure that your ethics statement is included in your manuscript, as the ethics statement entered into the online submission form will not be published alongside your manuscript.

**Reply:** We mentioned the ethics statement only in the method section.

8. We note that your Data Availability Statement is currently as follows: "All relevant data are within the manuscript and its Supporting Information files."

Please confirm at this time whether or not your submission contains all raw data required to replicate the results of your study. Authors must share the “minimal data set” for their submission. PLOS defines the minimal data set to consist of the data required to replicate all study findings reported in the article, as well as related metadata and methods (https://journals.plos.org/plosone/s/data-availability#loc-minimal-data-set-definition).

For example, authors should submit the following data:

- The values behind the means, standard deviations and other measures reported;

- The values used to build graphs;

- The points extracted from images for analysis.

Authors do not need to submit their entire data set if only a portion of the data was used in the reported study.

If your submission does not contain these data, please either upload them as Supporting Information files or deposit them to a stable, public repository and provide us with the relevant URLs, DOIs, or accession numbers. For a list of recommended repositories, please see https://journals.plos.org/plosone/s/recommended-repositories.

If there are ethical or legal restrictions on sharing a de-identified data set, please explain them in detail (e.g., data contain potentially sensitive information, data are owned by a third-party organization, etc.) and who has imposed them (e.g., an ethics committee). Please also provide contact information for a data access committee, ethics committee, or other institutional body to which data requests may be sent. If data are owned by a third party, please indicate how others may request data access.

**Reply:** We uploaded the original data and statistics as Supporting Information files.

Reviewers' comments:

Reviewer's Responses to Questions

**Comments to the Author**

1. Is the manuscript technically sound, and do the data support the conclusions?

The manuscript must describe a technically sound piece of scientific research with data that supports the conclusions. Experiments must have been conducted rigorously, with appropriate controls, replication, and sample sizes. The conclusions must be drawn appropriately based on the data presented.

Reviewer #1: Partly

Reviewer #2: Yes

---------------------------------------------------------------------------------------------------

2. Has the statistical analysis been performed appropriately and rigorously?

Reviewer #1: Yes

Reviewer #2: Yes

---------------------------------------------------------------------------------------------------

3. Have the authors made all data underlying the findings in their manuscript fully available?

The PLOS Data policy requires authors to make all data underlying the findings described in their manuscript fully available without restriction, with rare exception (please refer to the Data Availability Statement in the manuscript PDF file). The data should be provided as part of the manuscript or its supporting information, or deposited to a public repository. For example, in addition to summary statistics, the data points behind means, medians and variance measures should be available. If there are restrictions on publicly sharing data—e.g. participant privacy or use of data from a third party—those must be specified.

Reviewer #1: Yes

Reviewer #2: Yes

---------------------------------------------------------------------------------------------------

4. Is the manuscript presented in an intelligible fashion and written in standard English?

PLOS ONE does not copyedit accepted manuscripts, so the language in submitted articles must be clear, correct, and unambiguous. Any typographical or grammatical errors should be corrected at revision, so please note any specific errors here.

Reviewer #1: Yes

Reviewer #2: Yes

---------------------------------------------------------------------------------------------------

5. Review Comments to the Author

Reviewer #1: The present manuscript focuses on the effects of glycyrrhizin (GL) on inflammation and carcinogenesis in a murine model of colorectal cancer (CC). Wang and colleagues examine the role of regulatory T cells (Tregs) in this process and the potential mechanisms by which GL exerts its effects. The authors conducted experiments using ICR mice divided into four groups: control, GL alone, CC alone, and CC with GL treatment (CC+GL). After inducing colorectal cancer using azoxymethane (AOM) and dextran sodium sulfate (DSS), they measured various parameters such as spleen weight, white and red pulp areas, as well as different immune cell populations in splenic and colorectal tissues. The study reveals that during AOM/DSS-induced colon carcinogenesis, spleen enlargement and accumulation of Tregs and CD8+ cells (CTL) occur through the HMGB1-TLR4/RAGE NF-κB signaling pathway. GL inhibits these signaling pathways by targeting HMGB1, potentially impeding carcinogenesis processes.

However, the following issues must be addressed.

**Reply:** First of all, we would like to express our deep gratitude for your critical comments and kind advice, especially for comment #5 " They could have quantified the intensity of staining and normalized the total area, potentially avoiding human errors and bias." We quantified again using BZ-X800 Analyzer software (Ver. 1.1.10, KEYENCE, Osaka, Japan) to estimate the IHC staining intensity and normalize the total area as a percentage of the IHC staining positive area. The results by quantitative analyses are similar to the results from semi-quantitative analyses by IHC score. We changed all graphs of IHC score to IHC positive area percentage, to avoid human errors and bias.

1. Did the authors measure the CD4 T cell population in the red pulp? Does GL affect CD4 T effector cells in CC and CC+GL mice? If the authors had used flow cytometry, they might have obtained more precise quantitative data regarding immune cell populations. This represents one of the limitations of the study.

**Reply:** For this study, we used immunohistochemical analyses because of the limited sample number. Therefore, we could not try flow cytometry for checking the immune cell population including CD4-positive cells. We mentioned about it as the limitations of this study in the discussion part (Lines 372-379).

2. GL treatment alone increases the basal levels of CD8 and CD11c in the spleen and Foxp3 and RAGE expression in tumor tissue. Although the authors noted weak staining, could this be due to an overdose or potential side effects of GL? Explain.

**Reply:** Our new quantitative data showed CD8 positive area percentage in WP of GL group was significantly higher than that of the Control group, although there were no significant increases of other markers in GL group. Only by the above data it is difficult to explain it as an overdose or potential side effects of GL.

3. Is there any difference in the expression of FoxP3 and RAGE in the CC+GL mice group between CC1 and CC2 regions?

**Reply:** CC1 is the non-cancer cells surrounding to cancer cells (CC2). There was no significant difference between CC1 and Control group in the expression of FoxP3 and RAGE. On the other hand, their expressions in CC1 region were significantly lower than that of CC2 region. Therefore, CC1 region in CC group may be comparable to normal colon tissues.

4. The method section is not clearly written. How do the authors normalize spleen weights? Do they use tibia lengths?

**Reply:** We did not measure tibia lengths, and therefore we could not normalize spleen weight. However, there was no significant difference of body weight between four groups. Since there was no difference in the body weight of the mice, it is presumed that there was a significant difference in spleen weight of CC group.

We mentioned as below: “We did not measure tibia lengths to normalize spleen weight. However, there was no significant difference of body weight between four groups (Supporting Information files). Therefore, it is presumed that spleen weight can be assessed without normalization.” (Lines 98–100)

5. Throughout the manuscript, the authors measured cell counts or semi-quantitative staining grading to assess immune cell populations in IHC staining. They could have quantified the intensity of staining and normalized the total area, potentially avoiding human errors and bias.

**Reply:** Thank you very much your valuable advice. We quantified again using BZ-X800 Analyzer software (Ver. 1.1.10, KEYENCE, Osaka, Japan) to estimate the IHC staining intensity and normalize the total area as a percentage of the IHC staining positive area. We changed all graphs of IHC score to IHC positive area percentage, to avoid human errors and bias.

6. While the study suggests mechanisms involving Tregs and HMGB1, more detailed mechanistic studies are needed to fully understand GL's effects.

**Reply:** As you pointed, more detailed mechanistic studies are needed to fully understand GL's effects. Therefore, we described it in the discussion part, as the limitations of this study.

7. Understanding the effects of GL and Tregs in other tissues related to colorectal cancer progression would provide a more comprehensive picture.

**Reply:** To provide a more comprehensive picture to draw the relation of Tregs in other tissues and colorectal cancer and GL effects, many studies, which can elucidate mechanisms, are needed in the future.

8. Using additional colorectal cancer models, such as genetically engineered models or patient-derived xenografts, would provide a more comprehensive understanding of GL's potential effects across different cancer contexts.

**Reply:** We agree with your comment that colorectal cancer models, such as genetically engineered models or patient-derived xenografts, are the valuable methods to provide a more comprehensive understanding of GL's potential effects.

9. Further studies are needed to determine the clinical relevance of these findings in human colorectal cancer patients. The authors should mention the limitations of the manuscript.

**Reply:** Thank you for your comment. We described the limitations of this study in the discussion part.

10. Authors must thoroughly check their manuscript for typos and spacing errors.

**Reply:** We corrected typos and spacing errors throughout the manuscript.

Reviewer #2: Authors in this manuscript discuss most possible mechanisms by which Glycyrrhizin might suppress colon cancer development. The study focuses on the role of inflammation and immune response in the process.

The authors made a good colon cancer mice model via Inflammation induced by chemicals (AOM/DSS) damages DNA and promotes colon cancer. Resulting damage triggers a chain reaction involving molecules like NO, HMGB1, and signaling pathways.

Splenomegaly phenotype is seen due to the accumulation of immune cells CTLs, Tregs, DCs responding to inflammation and potential cancer.

Glycyrrhizin appears to disrupt this process by binding to HMGB1 and potentially affecting other signaling pathways, ultimately suppressing Tregs and cancer cell growth.

The data highlights the role of specific molecules HMGB1, NO and immune cells CTLs, Tregs in the process. Mechanism of GL's anticancer effect is proposed with supporting evidence from previous studies and present work.

The exact mechanism by which GL suppresses STAT3 and FoxP3 needs further investigation.

Overall, the manuscript provides a reasonable explanation of a potential mechanism for GL's anticancer properties. It highlights the complex interplay between inflammation, immune response, and cancer development. Further research is needed to validate the proposed mechanism fully. Though not completely new, this study adds a decent piece of knowledge to the existing literature on Glycyrrhizin`s possible mechanism of action.

**Reply:** We deeply appreciate your favorable comments.

---------------------------------------------------------------------------------------------------

6. PLOS authors have the option to publish the peer review history of their article (what does this mean?). If published, this will include your full peer review and any attached files.

If you choose “no”, your identity will remain anonymous but your review may still be made public.

Do you want your identity to be public for this peer review? For information about this choice, including consent withdrawal, please see our Privacy Policy.

Reviewer #1: Yes: Suman Asalla

Reviewer #2: No

[NOTE: If reviewer comments were submitted as an attachment file, they will be attached to this email and accessible via the submission site. Please log into your account, locate the manuscript record, and check for the action link "View Attachments". If this link does not appear, there are no attachment files.]

While revising your submission, please upload your figure files to the Preflight Analysis and Conversion Engine (PACE) digital diagnostic tool, https://pacev2.apexcovantage.com/. PACE helps ensure that figures meet PLOS requirements. To use PACE, you must first register as a user. Registration is free. Then, login and navigate to the UPLOAD tab, where you will find detailed instructions on how to use the tool. If you encounter any issues or have any questions when using PACE, please email PLOS at figures@plos.org. Please note that Supporting Information files do not need this step.

In compliance with data protection regulations, you may request that we remove your personal registration details at any time. (Remove my information/details). Please contact the publication office if you have any questions.
